# Interdisciplinary Collaboration in Head and Neck Cancer Care: Optimizing Oral Health Management for Patients Undergoing Radiation Therapy

**DOI:** 10.3390/curroncol31040155

**Published:** 2024-04-07

**Authors:** Tugce Kutuk, Ece Atak, Alessandro Villa, Noah S. Kalman, Adeel Kaiser

**Affiliations:** 1 Department of Radiation Oncology, Miami Cancer Institute, Baptist Health South Florida, Miami, FL 33176, USA; tugcek@baptisthealth.net (T.K.);; 2 Department of Radiation Oncology, Akdeniz University Faculty of Medicine, Antalya 07070, Turkey; eceatak.md@gmail.com; 3 Oral Medicine and Oral Oncology, Miami Cancer Institute, Baptist Health South Florida, Miami, FL 33176, USA; alessandro.villa@baptisthealth.net; 4 Department of Radiation Oncology, Herbert Wertheim College of Medicine, Florida International University, Miami, FL 33199, USA

**Keywords:** oral oncology, radiotherapy, head and neck cancer, dental management

## Abstract

Radiation therapy (RT) plays a crucial role in the treatment of head and neck cancers (HNCs). This paper emphasizes the importance of effective communication and collaboration between radiation oncologists and dental specialists in the HNC care pathway. It also provides an overview of the role of RT in HNC treatment and illustrates the interdisciplinary collaboration between these teams to optimize patient care, expedite treatment, and prevent post-treatment oral complications. The methods utilized include a thorough analysis of existing research articles, case reports, and clinical guidelines, with terms such as ‘dental management’, ‘oral oncology’, ‘head and neck cancer’, and ‘radiotherapy’ included for this review. The findings underscore the significance of the early involvement of dental specialists in the treatment planning phase to assess and prepare patients for RT, including strategies such as prophylactic tooth extraction to mitigate potential oral complications. Furthermore, post-treatment oral health follow-up and management by dental specialists are crucial in minimizing the incidence and severity of RT-induced oral sequelae. In conclusion, these proactive measures help minimize dental and oral complications before, during, and after treatment.

## 1. Introduction

Head and neck cancers (HNCs) account for approximately 67,000 new cancer diagnoses in the United States each year [1]. HNCs encompass cancers of various anatomic regions including the oral cavity, larynx, hypopharynx, nasopharynx, oropharynx, paranasal sinuses, and salivary glands (Table 1). The treatment of HNCs necessitates a multidisciplinary approach, with the majority of patients undergoing radiotherapy (RT) as an integral part of their treatment [2]. RT is a frequently utilized treatment modality in early-stage HNCs, either as a monotherapy or in conjunction with concurrent chemotherapy, serving as a definitive means of preserving organ function or as an adjuvant treatment following surgery in cases of locally advanced disease. Additionally, RT is utilized for palliating metastases to head and neck sites [3]. Notably, in select cases, RT produces superior functional outcomes compared to surgery [4]. The choice of the preferred RT modality for treatment may be influenced by various factors, including tumor location, treatment objectives, and tumor stage. The primary modalities utilized in HNC treatment encompass external beam radiotherapy (EBRT) and brachytherapy, with this paper primarily focusing on the more widely used EBRT. 

Specialized education and training are imperative for dental specialists to manage patients undergoing RT. Equipped with this expertise, specialists can effectively address the unique challenges of RT, deliver tailored preventive care, and manage oral treatment-related complications. This specialized knowledge ensures that patients receive comprehensive dental care precisely tailored to their individual needs. The aim of this review paper is to provide insight into the interdisciplinary collaboration involved in managing the dental aspects of patients with HNCs undergoing RT. 

## 2. Material and Methods

This paper is a clinical practice review, aiming to provide insight into the interdisciplinary collaboration required in managing the dental aspects of patients with HNCs undergoing RT. To gather articles for our paper, we employed a narrative literature search method utilizing the PubMed, Google Scholar, and ClinicalTrials.gov search engines. The selection process involved consensus among all authors, with a focus on identifying articles with potential benefits for clinical practice. Our search terms included ‘dental management’, ‘oral oncology’, ‘head and neck cancer’, and ‘radiotherapy’. We included studies, review articles, and editorials published in English between 1 January 2000 and 1 November 2023. Additionally, all obtained publications underwent a thorough review, and their key references were cross-checked to ensure a comprehensive and high-quality review. 

## 3. Radiation Therapy

### 3.1. Concept of Radiation Therapy

Ionizing radiation, employed in the treatment of cancer, transfers energy to tissues and generates free radicals that, in turn, cause single-strand and double-strand breaks in DNA, subsequently triggering apoptosis and promoting tumor regression [5]. While RT predominantly targets rapidly dividing cancer cells, it can also inadvertently harm adjacent normal tissues [6]. As a result, proactive strategies to mitigate the normal tissue effects are necessary during and after RT.

### 3.2. Radiation Therapy Techniques

The majority of HNCs are typically treated with EBRT, which employs high-energy photons (i.e., X-rays), electrons, or protons. Among EBRT techniques, intensity-modulated radiation therapy (IMRT) currently serves as the baseline standard for delivering radiation for curative HNCs [7]. IMRT involves ‘inverse’ planning to optimize beam angles and modulation, thereby maximizing the radiation dose to the target tissue while minimizing exposure to healthy surrounding tissue [8]. In practice, photon therapy is the most utilized form of RT for primary HNCs, but there is an increasing interest in proton beam therapy as a potentially less toxic treatment for HNCs [9]. Protons have a unique dose distribution, their entrance dose is minimal, and normal tissues beyond the specified target receive little to no exit dose; this type of treatment is therefore favored when treating deep-seated tumors that require a high dose of radiation but are located near radiosensitive normal tissues [10]. The dosimetric benefits of proton beam therapy are well-suited to unilateral treatments, effectively minimizing unnecessary radiation exposure to the midline and contralateral organs at risk while precisely conforming the dose to complex anatomical structures. This approach reduces the potential for iatrogenic toxicities. An increasing body of both retrospective and prospective studies has emerged, highlighting the evident dosimetric and clinical advantages associated with proton beam therapy [9,10]. Table 2 summarizes the radiotherapy techniques and their strengths and limitations.

Despite the proven superiority of proton therapy in achieving precise dose distributions and minimizing toxicity, its utilization remains low, with only 1% of newly diagnosed cancer cases in the US receiving proton therapy in 2018 [11,12,13]. However, certain cancer sites have demonstrated clear benefits from proton treatment, leading to its adoption as a standard indication in many countries [14,15]. Proton arc therapy has emerged as a promising modality, enabling treatment plan optimization with increased gantry angles compared to intensity-modulated proton therapy (IMPT). Recent studies have demonstrated its potential to further reduce the radiation dose to organs at risk and the normal tissue complication probability in HNC patients compared to traditional IMPT techniques [16].

### 3.3. Treatment Steps

**Figure 1:**
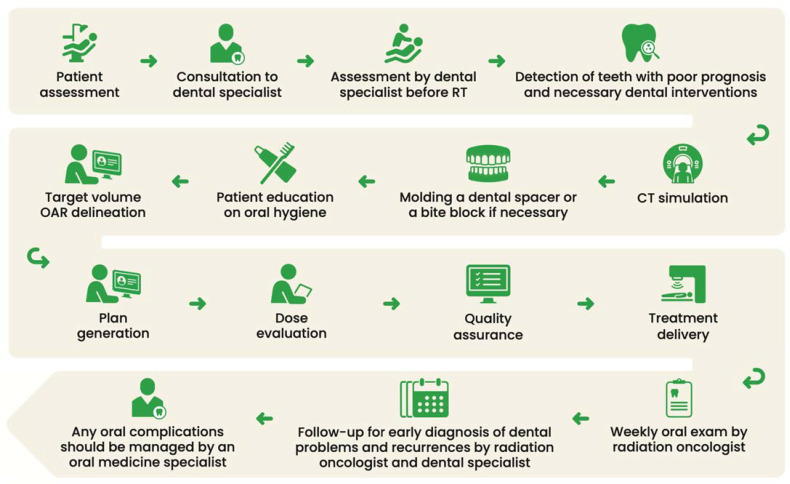
Head and neck cancer radiotherapy workflow and integration with dental/oral medicine specialist collaboration.

Following the initial consultation, patients progress through a series of steps, including evaluation, simulation, treatment planning, and actual treatment delivery, ultimately entering the post-RT follow-up phase. (Detailed steps are elucidated in Figure 1).

#### 3.3.1. Pre-Radiation Therapy Patient Evaluation

When an HNC patient is referred to a radiation oncology clinic, it is essential to further refer them to a dental specialist for a comprehensive oral evaluation, which should include intraoral radiographs or a panoramic radiograph, as recommended by the specialist, in preparation for RT. Teeth with an unfavorable long-term prognosis, such as those affected by severe dental caries or periodontal disease, may require extraction as part of the preparation for RT [17]. It is important to note that dentoalveolar surgery within the RT portals is a recognized risk factor for the development of osteoradionecrosis (ORN) of the jaws [18]. According to the National Comprehensive Cancer Network^®^ (NCCN) guidelines, it is advised to perform extractions of teeth with poor long-term prognoses at least 2 weeks before the initiation of RT to allow for primary healing [19]. Nevertheless, a recent study by Lee et al. suggests that treatment should not be delayed if postponing RT could have a detrimental effect on oncologic outcomes, given the low incidence of ORN resulting from pre-RT extractions [20]. Additionally, the removal of metallic dental restorations (MDRs) may serve as an effective strategy for mitigating the development of oral mucositis induced by backscatter emanating from MDRs during the course of RT [21]. Above all, patient education regarding proper oral hygiene assumes a pivotal role in preventing dental caries. Patients should receive guidance to use a prescription fluoridated toothpaste to brush teeth after each meal for dental caries prevention [22]. Table 3 provides an overview of the key recommendations for the dental evaluation of head and neck cancer patients before RT.

#### 3.3.2. Patient Simulation

**Figure 2:**
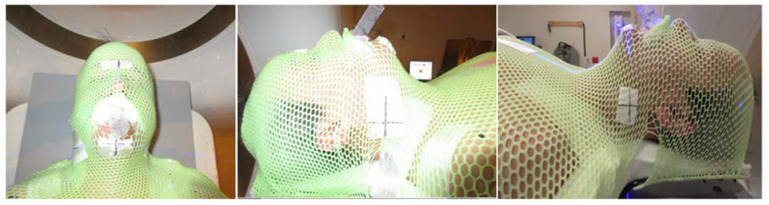
Example of a thermoplastic mask and bite block.

For modern RT planning, it is essential to perform computed tomography (CT) simulation, during which the patient is immobilized using an aquaplastic mask to minimize intra-fraction motion (Figure 2). To ensure the consistent positioning of oral cavity structures, particularly the tongue, for each RT fraction, simulation equipment such as bite blocks is employed. These bite blocks not only stabilize the tongue but also create a gap between the tongue and the roof of the mouth to reduce palatal dose exposure during the treatment of oral cavity and oropharynx lesions. For optimal fit and patient comfort during treatments, these devices are ideally custom-fabricated by a dental specialist. Furthermore, the radiation oncology team may request the dental provider to create custom intraoral RT prosthetics, including shielding prostheses, radiation carriers, positioning stents, or radiation mouth guards, to facilitate oncologic therapy and mitigate treatment-related toxicities on a case-by-case basis [23]. Dental spacers are an important tool in ensuring precise RT delivery by limiting radiation exposure to normal oral cavity tissues, consequently reducing the weight loss associated with mucositis-induced odynophagia [24,25]. In the context of metal amalgams or implants, the application of magnetic artifact reduction (MAR) methods can significantly enhance the quality of CT images. Corrupted CT images can have a profound impact not only on the precise delineation of the target area but also on the accuracy of dose calculations and Hounsfield unit (HU) measurements. When proton beams are employed in RT, even minor inaccuracies in tissue density can exert a considerable influence on the overall quality of treatment [26].

#### 3.3.3. Radiation Therapy Planning

**Figure 3:**
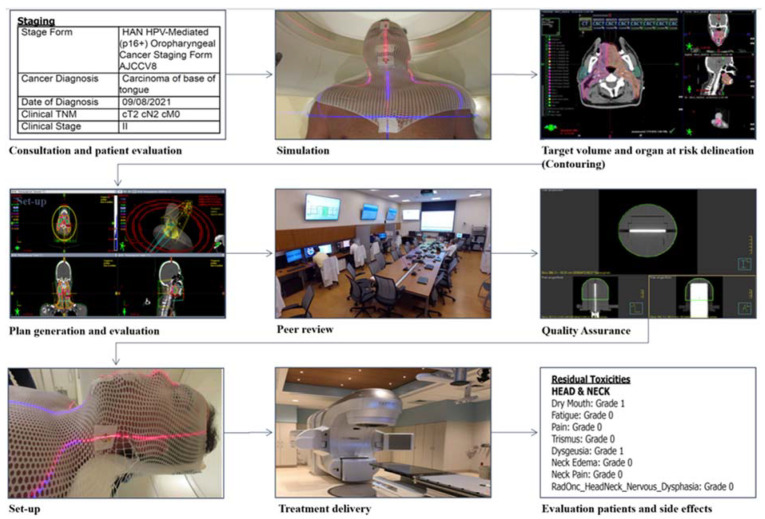
Radiation therapy steps for an example case of oral cavity cancer.

**Figure 4:**
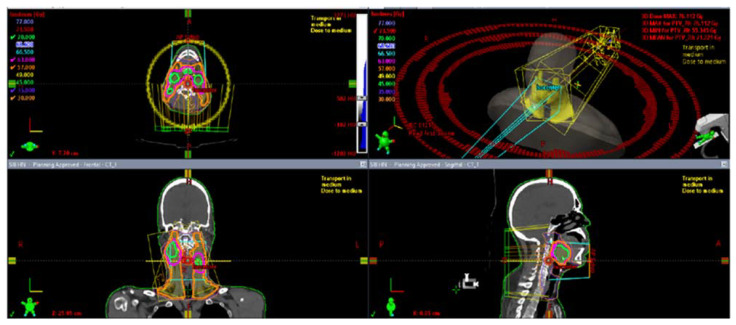
IMRT plan images for an example case of oral cavity cancer (isodoses—orange: 57 Gy, pink: 63 Gy, green: 70 Gy).

Using the simulation CT, radiation oncologists delineate target volumes and organs at risk (OARs) to enable the computerized optimization of radiation beams for maximizing tumor dose while minimizing OAR doses (Figure 3 and Figure 4) [27]. The gross tumor volume (GTV) contains the macroscopic primary tumor mass as well as the grossly involved neck lymph nodes. The clinical target volume (CTV) includes the tissues that are suspected of being infiltrated by microscopic tumor cells. A planning target volume (PTV) expansion is placed around the outlined targets to account for daily setup variations that are typically within 3–5 mm for head and neck patients treated with aquaplast mask immobilization [28]. Selecting the appropriate RT technique and conducting a meticulous plan evaluation are essential steps to prevent both acute and chronic side effects by limiting doses to the OARs. 

#### 3.3.4. Radiation Therapy Delivery

Once the RT plan is finalized, RT is initiated with the patient in the same position as during the simulation, and daily positioning accuracy is verified using CT or X-ray imaging. As the precision of treatment relies on reproducible positioning, weight loss resulting from RT-induced oral cavity side effects can create gaps between the patient and the aquaplastic masks, potentially leading to variations in positioning and suboptimal RT delivery. In these cases, adaptive RT offers notable advantages by accommodating anatomical changes, thereby reducing the doses received by adjacent healthy structures like parotid glands and enhancing the precision in target coverage [29,30].

## 4. Treatment-Related Side Effects and Management

The utilization of IMRT in the treatment of HNCs has contributed to reduced toxicity and enhanced quality of life compared to conventional techniques, primarily due to its capacity to more effectively spare critical OARs [10,23]. Notwithstanding these technological advancements, the occurrence of side effects remains a significant source of morbidity among patients undergoing treatment for HNCs [31]. The likelihood and severity of these side effects hinge on various factors, including the total radiation dose, fractionation, the concurrent chemotherapy regimen, and the specific anatomical regions targeted by the RT.

### 4.1. Management of Acute Side Effects during Radiation Therapy

Despite the diligent implementation of the prevention measures discussed earlier, it is essential to acknowledge that side effects during RT for HNCs are often inevitable. Among the acute side effects that may manifest during the RT course are fatigue, oral mucositis, alterations in taste, decreased appetite, dry mouth/thickened saliva, difficulties in swallowing, skin redness, burning sensations, pain in the treated area, breathing challenges, and consequent weight loss. Notably, two of the most frequently encountered and manageable dental side effects in this context are oral mucositis (OM) and dry mouth. 

#### 4.1.1. Oral Mucositis

Most patients undergoing curative RT for HNCs inevitably experience OM, characterized by painful erythematous and ulcerative lesions in the oral mucosa, often leading to taste alterations, swallowing difficulties, and increased susceptibility to infections [32]. RT causes continuous cellular damage to the epithelium due to the typical 2 Gy daily fractionated administration of irradiation from Monday to Friday until the cumulative dose reaches the prescribed treatment dose, usually around 70 Gy [33]. RT-induced OM begins to develop around the 10-day mark, when the cumulative radiation doses reach 15 Gy, with its severity intensifying around 30 Gy and potentially persisting for several weeks or even months [34]. The concurrent use of chemotherapy and tobacco consumption can elevate both the incidence and severity of OM, underscoring the importance of smoking cessation as a means of mitigating OM [35]. Patients are advised to avoid potential irritants, including alcohol-based mouthwashes and strongly flavored or spicy foods, all of which can exacerbate OM. To minimize oral mucosal trauma, a diet of soft, easy-to-chew and swallow foods is recommended. In 2020, the Multinational Association of Supportive Care in Cancer (MASCC) and the International Society of Oral Oncology (ISOO) jointly published a comprehensive guide for managing mucositis arising from cancer treatments. The implementation of multiagent combination oral care protocols was considered beneficial for preventing OM during HNC RT. However, due to limited data, the use of saline or sodium bicarbonate rinses in the prevention or treatment of OM could not be established. The expert panel recommended avoiding the use of chlorhexidine for OM prevention during RT [36]. Benzydamine mouthwash was recommended specifically in patients receiving moderate-dose RT (<50 Gy) and was also suggested for those undergoing RT combined with chemotherapy [37,38,39]. Intraoral photobiomodulation therapy, particularly low-level laser therapy, was also recommended for prevention during RT, regardless of chemotherapy involvement. Additionally, the panel suggested using a topical morphine 0.2% mouthwash for treating OM-associated pain in undergoing combined RT and chemotherapy. Conversely, sucralfate, whether applied topically or administered systemically, was not recommended for prevention in these patients. Oral glutamine was suggested for OM prevention in patients receiving combined RT and chemotherapy, while honey was suggested for those undergoing either RT alone or combined with chemotherapy [36]. Yet, effective treatments must address the treatment of OM; due to unmet clinical needs, various small molecules are in development, including avasopasem, bromonitrozidine, MIT-001, EC-18, ST-617, tempol, validive, and AG013 [33]. 

**Figure 5:**
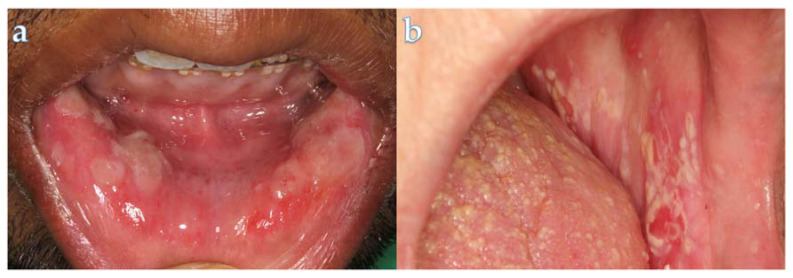
This figure illustrates two significant oral complications that can occur following radiotherapy for head and neck cancer: (**a**) oral mucositis and (**b**) oral pseudomembranous candidiasis.

In Figure 5, we illustrate two significant oral complications that can occur following RT for HNC: oral mucositis and oral pseudomembranous candidiasis.

#### 4.1.2. Oral Fungal Infections

Secondary bacterial and fungal infections are common, particularly in instances of suboptimal oral hygiene. The most frequent fungal infection is oral candidiasis, characterized by the manifestation of pseudomembranes and erythematous plaque [40]. This condition can adversely affect nutrition. Topical antifungal agents stand out as the preferred treatment option for mild cases of oral candidiasis. For more severe cases, fluconazole can be used as a systemic agent [41].

#### 4.1.3. Dry Mouth

Radiation-induced damage to the salivary glands can result in reduced saliva secretion, leading to the condition known as dry mouth, which encompasses both xerostomia (the subjective feeling of oral dryness) and hyposalivation (the objective reduction in salivary flow). Dry mouth is not only uncomfortable for the patient but can also bring about alterations in taste perception and difficulties with swallowing, speaking, and chewing [42]. Beyond the immediate inconveniences, hyposalivation elevates the risk of dental caries and oral candidiasis [43]. 

The recent ISOO/MASCC/ASCO guideline recommends choosing modalities that limit doses to salivary glands as a preventive measure [42]. To preserve salivary gland function, a critical measure involves limiting radiation doses to protect their functionality. The introduction of IMRT has significantly improved HNC outcomes by enabling radiation techniques that spare the parotid glands, resulting in a clinically meaningful reduction in both the incidence and severity of dry mouth [44]. By maintaining the TD50 (the dose at which 50% of patients experience toxicity) below 39 Gy, the risk of dry mouth can be reduced to less than 25% [45]. Current efforts to improve radiotherapy planning and reduce xerostomia involve strategies such as field reduction, particle therapy, and dose de-escalation. Field reduction specifically means reducing planning margins and excluding certain levels of neck coverage as a preventive measure. The pursuit of refining the preservation of salivary gland tissue has seen significant progress through innovative radiation techniques utilizing protons. Protons’ unique physical and radiobiological properties enable a superior dose distribution, concentrating the maximum deposited energy dose with greater precision. Comparative analyses have consistently demonstrated the potential advantages of proton therapy over conventional photon methods, emphasizing its efficacy in minimizing radiation exposure to normal tissues [46,47,48,49]. 

Upon defining a subgroup of HPV-positive oropharyngeal cancer patients with a more favorable prognosis, researchers initiated an inquiry into the feasibility of dose de-escalation in selected cases [50]. Furthermore, with the FMISO adaptive protocols tracing hypoxia, dose de-escalation has the potential to achieve comparable treatment effectiveness while minimizing treatment-related toxicity [51].

When patients develop dry mouth after RT and aim to stimulate salivation, the most effective treatments are muscarinic agonists such as pilocarpine and cevimeline [52]. To reduce discomfort, mucosal lubricants and salivary substitutes can be prescribed. Moreover, sugar-free lozenges, chewing gums, and acupuncture are other treatment options [42,53]. 

### 4.2. Management of Post-Radiation Therapy Complications

Should patients develop dentoalveolar pathology following head and neck RT, it is important for the patient to consult the treating radiation oncology team prior to any surgical dental intervention. Given the site-specific effects of RT, a comprehensive review of RT records, including RT planning details and dose–volume histograms (DVHs), is essential (Figure 3).

Oral complications from HNC therapy are frequently managed by oral medicine specialists. For patients requiring dental prosthetics after treatment, it is advisable to seek care from an oral and maxillofacial prosthodontist. For general dental needs, patients can safely consult with a general dentist, provided there is effective communication with the treating oncologist during and following cancer therapy.

#### 4.2.1. Dental Caries

While many acute toxicities tend to subside after treatment, patients remain susceptible to long-term complications, particularly hyposalivation, following RT. Apart from the adverse impact on patient quality of life, hyposalivation is a recognized risk factor for the development of dental caries and oral candidiasis. The prevalence of caries among patients who have undergone RT stands at approximately 25% [54]. RT-associated caries tend to progress more rapidly and are more likely to affect non-classical tooth surfaces compared to classical caries. Additionally, these caries exhibit a higher rate of recurrence and an increased risk of dental treatment failure, often necessitating additional dental procedures [55]. The early detection of caries through multiple yearly dental visits and the prescription of high-strength fluoride are recommended to preserve oral health. 

#### 4.2.2. Periodontal Diseases

Irradiated periodontal tissues are notably more vulnerable to gingival recession and attachment loss, primarily attributable to heightened oxidative damage resulting from irradiation [56]. In this context, effective biofilm and plaque control play a pivotal role and, when periodontal interventions become necessary, a conservative approach is strongly recommended to mitigate the risk of ORN [57].

#### 4.2.3. Trismus

Trismus, characterized by restricted mouth opening, can be attributed to factors such as tumor infiltration into masticatory muscles, the temporomandibular joint (TMJ), or the trigeminal nerve, and it may manifest as an acute or a late side effect of radiotherapy, often resulting from the fibrosis of ligaments and muscles, leading to contracture [58]. Impaired opening of the jaw can result in difficulties with eating, speech, and maintaining oral hygiene and presents challenges during dental examinations and oncologic follow-ups [59]. When a patient presents with delayed trismus, it is crucial to determine whether it is a result of treatment side effects or a sign of tumor recurrence [60]. After the exclusion of recurrence, the main treatment option for management is conservative physical therapy such as mobilization exercises to improve the flexibility of TMJ and strengthen the muscles [60]. Botulinum toxin injections may help alleviate the pain caused by muscle spasms [61]; however, they do not improve mouth opening [61]. For selected patients who are non-responsive to other treatments, surgical interventions such as chordectomy can be another treatment option [62]. 

#### 4.2.4. Dysgeusia

The change in the perception of taste starts early during RT and reaches its peak around 60 Gy [63]. RT-induced dysgeusia happens due to alterations in the gustatory cells [64] The sensation of acidic and bitter flavors is impaired more rapidly than that of sweet and salty tastes [65]. These alterations are mostly reversible and typically return within one year after treatment; however, in some cases, dysgeusia can be permanent. Recent research suggests that proton therapy, by reducing the radiation dose to the tongue, not only shows lower rates of acute toxicities but also significantly mitigates dysgeusia during both subacute and chronic phases of treatment [46,49].

#### 4.2.5. Osteoradionecrosis

Osteoradionecrosis was previously defined as the ischemic necrosis of bones in conjunction with soft tissue necrosis, all in the absence of any tumor presence [66]. The underlying pathophysiology is not well established, but theories suggest it may involve hypovascular and hypoxic disturbances in wound healing, osteoclast damage, alterations in vascularity, fibroatrophia, endothelial dysfunction, and fibrosis [67]. Reports have indicated a variable incidence of ORN, ranging from 4% to 37%, with a decreasing risk observed in more recent studies thanks to advances in RT [40]. Areas previously subjected to high-dose RT, typically exceeding 60 Gy, are at an elevated risk of developing jaw ORN. Consequently, dentoalveolar surgeries, including dental extractions, dental implants, periodontal procedures, and deep cleanings, may not be recommended in consideration of this heightened risk [68]. Factors such as poor oral hygiene, the presence of ill-fitting dentures, or post-RT dentoalveolar surgeries can further increase the risk of ORN [69]. Notably, the occurrence of ORN following dental extraction is significantly higher in the mandible than in the maxilla, primarily attributed to the distinctive vascularization pattern of the mandible [68]. 

To mitigate the risk of ORN, it is recommended to address existing oral diseases and stabilize oral health both before and after RT. As a preventive measure, the use of pentoxyfylline in combination with tocopherol (vitamin E) before and after dentoalveolar interventions is advised [70,71]. Pentoxyfylline, a phosphodiesterase inhibitor, promotes vascularization, while tocopherol exhibits antioxidant properties [72]. While there is no universally established algorithm for the use of pentoxyfylline, most studies recommend daily dosages of 800 mg pentoxyfylline with 1000 IU of tocopherol for at least six months [71]. Additionally, adding Clodronate to the treatment has been shown to yield improved results [73]. In the management of ORN, proposed treatment protocols include conservative therapy involving antibiotic therapy and surgical resection and reconstruction for non-responsive cases [74]. The role of hyperbaric oxygen therapy in the context of dentoalveolar surgery remains a subject of controversy [75,76].

Overall, to address RT-related dental complications, it is important to understand their nature and severity and respective management approaches. Table 4 summarizes the most common side effects and effective strategies for their management.

## 5. Follow-Up Recommendations

Long-term daily fluoride supplementation has been recommended as a preventive measure to mitigate the risks of developing dental caries in the context of hyposalivation following radiotherapy, with various modes of fluoride supplementation proposed in the past [77]. Nevertheless, it is essential to consider patient compliance as a pivotal component of the final treatment strategy. Patients should also resume routine dental hygiene maintenance with their local general dental providers and may choose to schedule more frequent dental hygiene appointments to aid in the maintenance of their dentition. The collaboration between dental specialists and radiation oncologists is fundamental for comprehensive cancer care, particularly in managing potential dental complications, with a specific emphasis on post-RT dental follow-up during each subsequent visit. 

### Dental Implants Post Radiotherapy

The impact of RT on dental health, particularly in HNC patients, presents significant challenges and considerations for oral rehabilitation with dental implants. Tooth loss resulting from RT-induced side effects such as rampant caries, poor oral hygiene, and hyposalivation compromises patients’ oral function and quality of life. Dental implant-supported prostheses have emerged as a promising solution for HNC patients unable to wear conventional dentures due to RT-related side effects [78]. However, the potential risks associated with dental implant procedures following RT, including ORN and uncertainties regarding the optimal timing and procedural considerations for implant placement in patients undergoing or having undergone RT, remain areas of concern [79]. The invasive nature of dental implant surgery poses additional challenges, with jawbone trauma inevitable in such patients and the risk of ORN persisting for the patient’s remaining life [80,81]. As the majority of published systematic reviews focus solely on the timing of implant placement after RT, further research is warranted to address these challenges and improve the oral rehabilitation outcomes and quality of life for HNC patients.

## 6. Future Directions

Ensuring adequate training for dentists in managing the oral health needs of oncology patients undergoing HNC treatment is imperative. However, the existing literature underscores notable deficiencies within dental school curricula regarding radiation side effects and RT-related oral complications. Studies indicate significant variability in dentists’ knowledge levels concerning RT complications and their management, with some professionals lacking confidence in identifying early changes necessitating intervention [82,83,84]. Recommendations from dental associations like the British Association of Head and Neck Oncologists (BAHNO) and the International Society of Oral Oncology (ISOO) stress the importance of dentists treating HNC patients possessing accurate knowledge of illness burdens, prevention, and treatments of oral complications [36,85]. Nevertheless, surveys reveal widespread perceptions among dental students and practitioners of insufficient undergraduate education in HNC care, leading to notable deficits in clinical practice [82,83,84,86,87,88]. Additional training, as recommended by dental associations like the American Dental Association (ADA), Canadian Dental Association (CDA), and European Dental Association (EDA), is crucial to ensure that dentists possess adequate knowledge of RT side effects and are well prepared to care for HNC patients. This supplementary training should encompass specialized courses focusing on HNC therapy-related oral complications and their prevention and management [87,89]. Topics covered in these courses should include the identification and management of acute and late radiation side effects, such as mucositis, xerostomia, osteoradionecrosis, and dental caries. Furthermore, emphasis should be placed on techniques for oral examination and the assessment of HNC patients before, during, and after RT, along with strategies for interdisciplinary collaboration with oncology providers [87]. Encouraging ongoing professional development activities, workshops, and seminars can also ensure that dentists remain updated on the latest evidence-based practices in managing HNC patients’ oral health needs post-RT.

While many dental school curricula touch upon topics related to the oral health needs of HNC patients, the extent and depth of coverage vary significantly across institutions. Some dental schools may offer dedicated courses or modules on oncology dentistry, covering oral complications of cancer treatment, including RT [82,83,84]. However, the level of emphasis on oncology dentistry and RT-related complications can vary. Therefore, integrating these topics into broader courses on oral medicine, oral pathology, or oral and maxillofacial surgery should be supplemented by additional specialized training to ensure consistency in preparedness among dental students [36,85].

## 7. Conclusions

In conclusion, the management of HNC patients who have undergone RT necessitates a collaborative and dynamic approach involving radiation oncology and dental specialist teams. A clear understanding of the respective roles of each healthcare provider within the care team is essential to achieve this objective. Acute and long-term care strategies are jointly formulated, with the overarching goal of optimizing patient quality of life by minimizing complications arising from curative therapies. This interdisciplinary collaboration and a patient-centered approach are fundamental to ensuring comprehensive care for HNC patients throughout their treatment journey and beyond.

## Figures and Tables

**Table 1:** Localization of head and neck cancers.

Type of HNC	Regions Included
Oral Cavity Cancer	Lips, Buccal Mucosa, Gingiva, Tongue, Floor of Mouth, Retromolar Trigone, Hard Palatal mucosa
Oropharyngeal Cancer	Tonsils, Soft Palate, Base of Tongue, Posterior Pharyngeal Wall
Nasopharyngeal Cancer	Nasopharyngeal Tissue, Posterior Nasal Cavity
Sinonasal Cancer	Nasal Cavity, Paranasal Sinuses
Hypopharynx Cancer	Pyriform Sinus, Postcricoid Region
Larynx Cancer	Supraglottis, Glottis, Subglottis
Salivary Gland Cancer	Parotid Glands, Submandibular Glands, Sublingual Glands, Minor Salivary glands

**Table 2:** Radiation therapy techniques for head and neck cancers.

Technique	Description	Advantages	Disadvantages
**3D Conformal Radiation Therapy (3DCRT)**	Uses CT scans to shape radiation beams and conform them to the tumor’s geometry in the head and neck region	- Efficient for straightforward tumor shapes - Faster treatment planning - Lower cost compared to other techniques	- Higher doses to surrounding healthy tissues - Limited application for complex tumor shapes
**Intensity-Modulated Radiation Therapy (IMRT)**	Precise radiation delivery with varying intensities to conform to the tumor’s shape in the head and neck area	- Reduced dose to critical structures like salivary glands - Suitable for complex tumor shapes	- Complex treatment planning
**Proton Therapy**	Utilizes proton particles instead of X-rays for precise dose delivery, minimizing damage to surrounding tissues in head and neck cancers	- Superior healthy tissue sparing - Suitable for pediatric cases	- Limited availability of proton therapy centers - Higher cost
**Brachytherapy**	Involves the placement of radioactive sources directly into or near the tumor in the head and neck	- Reduced dose to surrounding tissues - Shorter overall treatment time	- Invasive procedure - Requires specialized expertise

**Table 3:** Pre-radiotherapy dental evaluation recommendations.

Recommendations	Educational Objectives
Consultation with a dental specialist	Gain insights into the importance of dental specialist consultations in assessing and planning treatment
Extractions of teeth with poor prognoses	Learn to identify and manage problematic teeth
Management of metallic dental restorations	Understand the significance of addressing metallic restorations in radiotherapy planning
Education for maintaining oral hygiene	Emphasize the role of proper oral hygiene in preventing radiation-related dental issues
Recommending prescription fluoride toothpaste	Explore the use of prescription fluoride toothpaste for dental caries prevention during and after treatment
Recommending smoking cessation	Highlight the importance of advising patients to quit smoking for better treatment outcomes
Implementation of dental spacers or prosthetics	Understand the use of dental spacers and prosthetics in protecting oral tissues during radiotherapy

**Table 4:** Recommendations for radiotherapy side effect management for dental specialists.

Recommendations	Educational Objectives
**Oral mucositis:**	
Preventing radiation-induced oral mucositis	Learn the oral care protocols for the prevention of oral mucositis and management during radiotherapy
Early recognition of radiation-induced oral mucositis by weekly oral examination	Understand the importance of timely detection and the role of regular examinations in patient care
**Oral infections:**	
Prescription of antibiotics and antifungals when needed	Explore the use of antibiotics and antifungals in treating radiation-induced infections
**Dry mouth:**	
Maintaining adequate hydration	Learn how hydration techniques alleviate dry mouth and prevent dental complications
Prescription of anticholinergic medications	Understand the pharmacological approach to stimulating saliva production in dry mouth cases
Close follow-up for increased risk of dental caries	Gain awareness of the monitoring process to prevent dental caries in patients with dry mouth
**Trismus:**	
Mobilization exercises to maintain jaw opening	Learn exercises to preserve jaw mobility during and after radiotherapy
Alleviating symptoms to improve quality of life	Learn the indications of analgesics, muscle relaxants, and anti-inflammatory medications
Referring to a surgeon for release surgery in advanced cases	Learn when surgical intervention becomes necessary for severe trismus and how to make appropriate referrals
**Osteoradionecrosis:**	
Avoiding unnecessary dental interventions	Understand the significance of minimizing oral surgical procedures to reduce the risk of osteoradionecrosis
Pentoxyfylline and tocopherol prophylaxis	Learn the use of prophylactic medications to decrease the likelihood of osteoradionecrosis
Antibiotic therapy when needed	Learn the role of antibiotics in treating infections associated with osteoradionecrosis and when they are required

## Data Availability

Research data are stored in an institutional repository and will be shared upon request to the corresponding author.
